# Anatomic Diagram as a Novel Assessment Strategy for Subclinical Local Residual Disease in Sinonasal Squamous Cell Carcinoma and Intestinal‐Type Adenocarcinoma

**DOI:** 10.1002/alr.70071

**Published:** 2025-11-28

**Authors:** Piergiorgio Gaudioso, Leonardo Calvanese, Stefano Taboni, Giacomo Contro, Diego Cazzador, Tommaso Saccardo, Gloria Schiavo, Vittorio Rampinelli, Alberto Schreiber, Gabriele Testa, Cesare Piazza, Enzo Emanuelli, Davide Mattavelli, Piero Nicolai, Marco Ferrari

**Affiliations:** ^1^ Department of Neuroscience (DNS) Section of Otorhinolaryngology ‐ Head and Neck Surgery, University of Padova Padova Italy; ^2^ Unit of Otorhinolaryngology ‐ Head and Neck Surgery Azienda Ospedale‐Università Padova Padova Italy; ^3^ Department of Surgery Oncology and Gastroenterology (DiSCOG) Oncology and Immunology (PhD Program), University of Padova Padova Italy; ^4^ Department of Surgery ENT Unit, Ospedali Riuniti Padova Sud Monselice PD Italy; ^5^ Department of Information Engineering Technology For Health (PhD Program), University of Brescia Brescia Italy; ^6^ Department of Clinical Neurosciences Clinic of Otorhinolaryngology‐Head and Neck Surgery, Geneva University Hospitals, University of Geneva Geneva Switzerland; ^7^ Department of Otolaryngology Head and Neck Surgery ASST Lariana, Ospedale Sant'anna University of Insubria Como Italy; ^8^ Unit of Otorhinolaryngology ‐ Head and Neck Surgery ASST Spedali Civili Di Brescia Brescia Italy; ^9^ Department of Medical and Surgical Specialties Radiological Sciences, and Public Health University of Brescia Brescia Italy; ^10^ Division of Otorhinolaryngology ASST Del Garda Presidio Ospedaliero di Manerbio Manerbio Italy; ^11^ Division of Otorhinolaryngology ASST Del Garda Presidio Ospedaliero di Desenzano del Garda Desenzano del Garda Italy; ^12^ Department of Neuroscience (DNS) Section of Otolaryngology University of Padova Treviso Italy

**Keywords:** anterior skull base, endoscopic minimally invasive surgery of the skull base, endoscopic skull base surgery, skull base

## Abstract

**Objective:**

In the last two decades, transnasal endoscopic surgery (TES) has become pivotal in the management of sinonasal tumors. This approach involves a multiblock tumor resection, adding complexity to the interpretation of surgical margins after pathological examination. This study compares different strategies to infer subclinical local residual disease (SLRD), aiming to identify and validate the best available method for assessing SLRD after transnasal endoscopic resection of sinonasal squamous cell carcinoma (SCC) and intestinal‐type adenocarcinoma (ITAC).

**Methods:**

Three methods to estimate SLRD (as either absent—R0 ‐ or microscopically present—R1) were applied in patients who received negative margins‐aimed endoscopic resection: sole‐pathologist examination, multidisciplinary evaluation, and anatomic diagram‐based assessment. The primary outcome to compare methods was time‐to‐recurrence (TTR) stratification provided by these methods.

**Results:**

105 patients were included (50 SCC and 55 ITAC). All three methods resulted significantly associated with TTR in both ITAC and SCC populations. In a multivariate model, only SLRD assessed with the anatomical diagram was independently associated with time‐to‐local‐recurrence (TTLR) in SCC and TTR in both ITAC and SCC groups. The concordance index (C‐index), the area under the curve (AUC), and the incremental AUC (iAUC) were higher for the anatomical diagram method in the ITAC and SCC cohorts.

**Conclusion:**

The anatomic diagram proved to be the best available strategy, yet with limitations, for assessing SLRD, demonstrating superior TTR stratification compared to traditional methods.

## 1 Introduction

Sinonasal malignancies account for 3%–5% of all head and neck cancers and exhibit marked histological heterogeneity. Squamous cell carcinoma (SCC) stands as the predominant variant, closely followed by intestinal‐type adenocarcinoma (ITAC) [1]. Surgery is the gold standard treatment when negative margins can be achieved [2], and is frequently followed by adjuvant radiotherapy (RT) [3]. The proximity of the tumor site to critical anatomic structures (such as the orbit and skull base) poses a considerable challenge in achieving complete resection, making the therapeutic endeavor particularly demanding [4].

Over the last two decades, transnasal endoscopic surgery (TES) has become pivotal in the management of these tumors, superseding anterior craniofacial resection techniques [5, 6, 7, 8]. Despite the recent literature showing comparable 5‐year survival rates between open surgery and TES, the former may entail a higher risk of functional and cosmetic morbidity [9, 10, 11, 12, 13, 14, 15]. When feasible, transnasal endoscopic resection is considered a safer surgical option in the management of sinonasal tumors with a growing body of evidence, owing to a lower rate of complications and non‐inferior oncologic outcomes compared with open approaches [16, 17]. On the other hand, TES requires a multiblock resection of the tumor [16], which increases the complexity of interpretation of surgical margins. Therefore, a comprehensive analysis of the resected tissues from a surgical and pathological standpoint is essential to understand the three‐dimensional extension of the tumor and to assess the presence of subclinical local residual disease (SLRD) [18, 19, 20]. This is relevant because presence of SLRD is associated with a reduction in survival probability [21]. Additionally, dose and volumes of adjuvant RT are adjusted based on several factors, including presence and localization of SLRD [16, 22, 23]. Interpretation of specimens resulting from transnasal endoscopic resection requires a mental topographic reconstruction of the resected blocks, differentiating between infiltrated and non‐infiltrated tissues, which can be laborious. Bastier and de Gabory [24] ideated a sinonasal anatomic scheme, designed to optimize the assessment of the size, location, and extension of sinonasal tumors relative to the ablated tissues, potentially improving interdisciplinary communication and refining understanding of tumor extension and SLRD.

**FIGURE 1:**
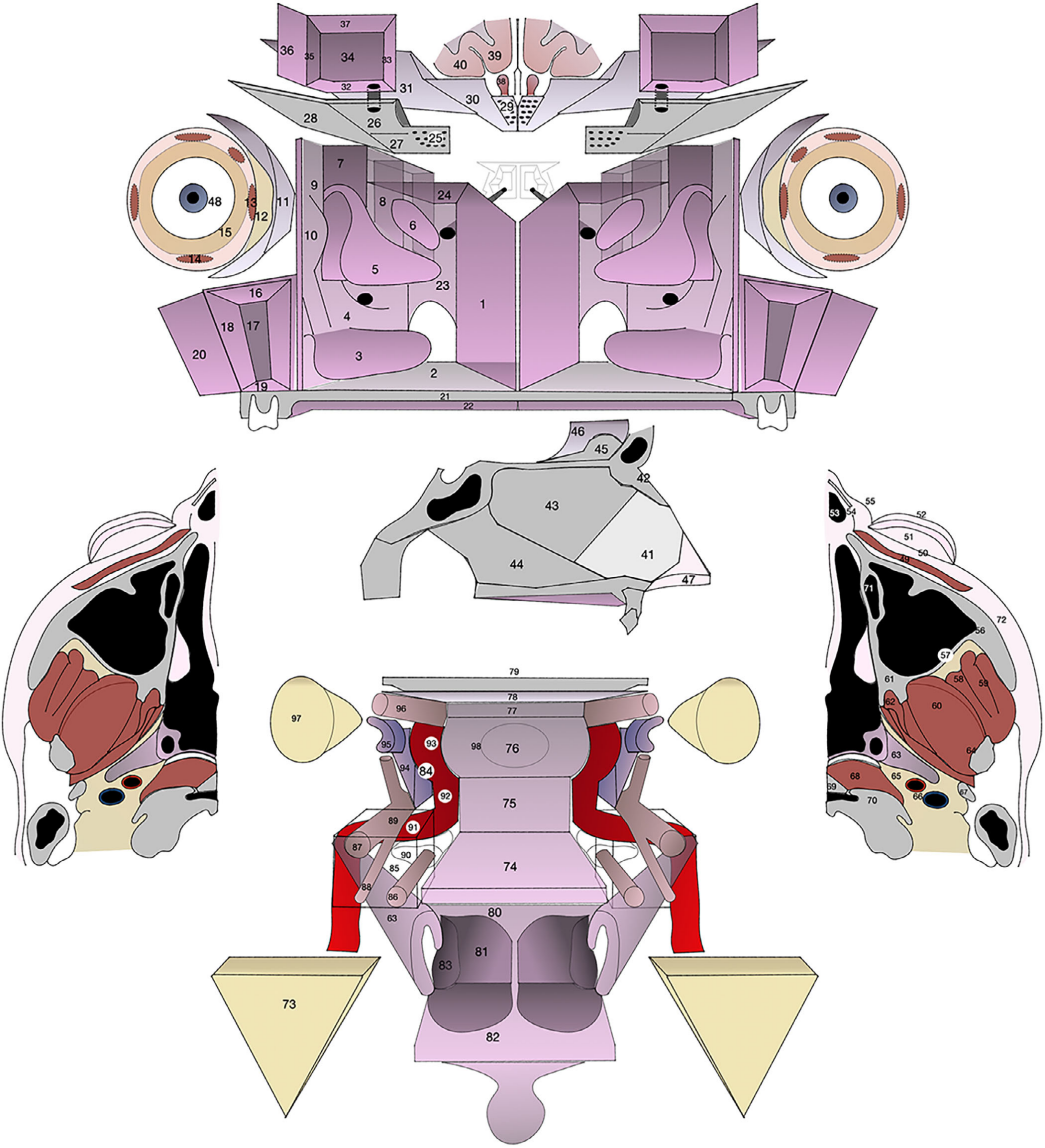
The modified and expanded version of the sinonasal anatomic diagram.

A modified version of the aforesaid anatomic diagram (Figure 1) is herein presented. This study aims to validate this method for interpretation of the SLRD after transnasal endoscopic resection, by comparing it with currently available strategies used to infer this information in patients affected by sinonasal cancers.

## 2 Materials and Methods

This study was performed in line with the principles of the Declaration of Helsinki. All patients signed a detailed informed consent form regarding the processing and publication of their data. Retrospective research on these patients was approved by the local institutional review boards (“Comitato Etico Territoriale Area Centro—EST Veneto”, code 433n/AO/23; “Comitato Etico di Brescia”, code NP‐3616). Data were examined in agreement with the Italian privacy and sensitive data laws, and the internal regulations of the Universities of Padova and Brescia.

### 2.1 Data Collection

The present study included patients diagnosed with primary or recurrent sinonasal SCC or ITAC who underwent upfront TES in the Section of Otolaryngology—Head and Neck Surgery of the University Hospitals “Azienda Ospedale Università di Padova” (Padova, Italy) and “Spedali Civili di Brescia” (Brescia, Italy) in the period from March 2007 to July 2021. Inclusion criteria comprised: (a) upfront transnasal endoscopic resection aiming at negative margins for sinonasal SCC or ITAC; (b) any stage; (c) any presentation (primary or recurrent tumors); a minimum follow‐up of 6 months. Endoscopic‐assisted or purely open resections, lack of relevant information in the surgical and pathological report, lack of postoperative multidisciplinary discussion, and a follow‐up shorter than 6 months were exclusion criteria.

The surgical treatment was aimed at achieving negative margins in all the included patients, who were treated according to the principles reported in the “Multi‐institutional study on endoscopically treated sinonasal cancers”[13] and followed in keeping with the “European position paper on endoscopic management of tumors of the nose, paranasal sinuses, and skull base”[14].

For all patients, both the pathological examination and the postoperative multidisciplinary evaluation were retrospectively reviewed. Subsequently, based on the structures found to be microscopically infiltrated at the pathological examination, a three‐dimensional map of tumor extension was reconstructed using the anatomic diagram as a reference (Figure 1). Each structure (Table 1) was classified as infiltrated, non‐infiltrated, or non‐analyzed (if resected but not included in the pathological examination). The number of analyzed subunits varied among cases and tumor sites, and no fixed minimum number of subunits was predefined. The protocol enabling the assessment of tumor extension in relation to resected anatomical subunits was based on two key principles: (1) additional resection of anatomical layers at the margins of the primary ablation was routinely undertaken when clinical judgment indicated a potential risk of tumor spread in a given area, provided that removal of macroscopically normal tissue would not result in unacceptable morbidity or morbidity beyond patient's consent; and (2) whenever feasible, anatomical subunits were identified and delineated within the main surgical specimen. Additional collected parameters were clinical stage, previous treatments, adjuvant therapy, and follow‐up.

**TABLE 1:** Anatomic structures included in the anatomic diagram.

Non‐Midline Structures
1 Septal mucosa
2 Floor mucosa
3 Inferior turbinate
4 Lateral nasal wall/Medial maxillary wall
5 Middle turbinate
6 Superior turbinate
7 Anterior ethmoidal complex
8 Posterior ethmoidal complex
9 Lamina papyracea
10 Lacrimal fossa/sac
11 Periorbit
12 Extraconal fat
13 Medial rectus muscle
14 Inferior rectus muscle
15 Intraconal fat
16 Roof of the maxillary sinus/orbital floor
17 Posterior maxillary wall
18 Lateral maxillary wall
19 Alveolar process
20 Anterior maxillary wall
21 Hard palate (bone)
22 Hard palate (soft tissues‐mucosa)
23 Anterior sphenoidal wall/sphenoidal rostrum
24 Olfactory mucosa
25 Cribriform plate
26 Anterior ethmoidal roof
27 Posterior ethmoidal roof
28 Orbital roof
29 Olfactory groove/dura
30 Dura of the ethmoidal roof
31 Dura of the orbital roof/posterior frontal plate
32 Floor of frontal sinus/frontal beak
33 Interfrontal sinus‐septum
34 Posterior frontal plate
35 Lateral recess of the frontal sinus/supraorbital cell
36 Anterior frontal plate
37 Frontal bone above frontal sinus
38 Olfactory bulb
39 Gyrus rectus
40 Medial orbital gyrus
48 Eyeball/optic nerve
49 Premaxillary periosteum/muscles
50 Premaxillary subcutaneous tissue /skin
51 Lower preseptal space /eyelid
52 Upper preseptal space/eyelid
53 Vestibular skin
54 External nose lateral cartilages
55 External nose skin
56 Zygomatic process/bone
57 Infratemporal fat pad
58 Temporal muscle
59 Coronoid process
60 Lateral pterygoid muscle
61 Pterygoid plates
62 Medial pterygoid muscle
63 Eustachian tube/tubal muscles
64 Condylar process (mandible)
65 Upper parapharyngeal space
66 Internal carotid artery (parapharyngeal) and internal jugular vein
67 Styloid process/muscles
68 Prevertebral muscles
69 Anterior arch of the atlas
70 Lateral mass of the atlas
71 Nasolacrimal duct
72 Zygomatic skin
73 Pterygopalatine fossa
74 Sphenoidal floor (bone)—lower clivus
75 Midclivus
76 Sellar prominence (bone)
77 Tuberculum sellae
78 Planum sphenoidale
79 Dura of the planum sphenoidale
80 Nasopharyngeal vault (soft tissue)
81 Posterior nasopharyngeal wall
82 Soft palate
83 Lateral recess of the nasopharynx
84 Lateral sphenoidal wall (bone)—Carotid prominence (bone)—Optic canal (bone)
85 Base of the pterygoid process
86 Vidian canal/nerve
87 Maxillary nerve/foramen rotundum
88 Mandibular nerve/foramen ovale
89 Gasserian ganglion/Meckel's cave
90 Fibrocartilago basalis
91 Internal carotid artery (petrous)
92 Internal carotid artery (paraclival)
93 Internal carotid artery (parasellar)
94 Cavernous sinus
95 Superior orbital fissure
96 Optic nerve
97 Orbital apex (posterior third)
98 Sella/Suprasellar region

Midline Structures
41 Septal cartilage (quadrangular plate)
42 Nasal bone /nasal dorsum skin
43 Perpendicular plate
44 Vomer
45 Crista galli
46 Falx cerebri
47 Columella‐membranous septum

### 2.2 Interpretation of Subclinical Local Residual Disease

**FIGURE 2:**
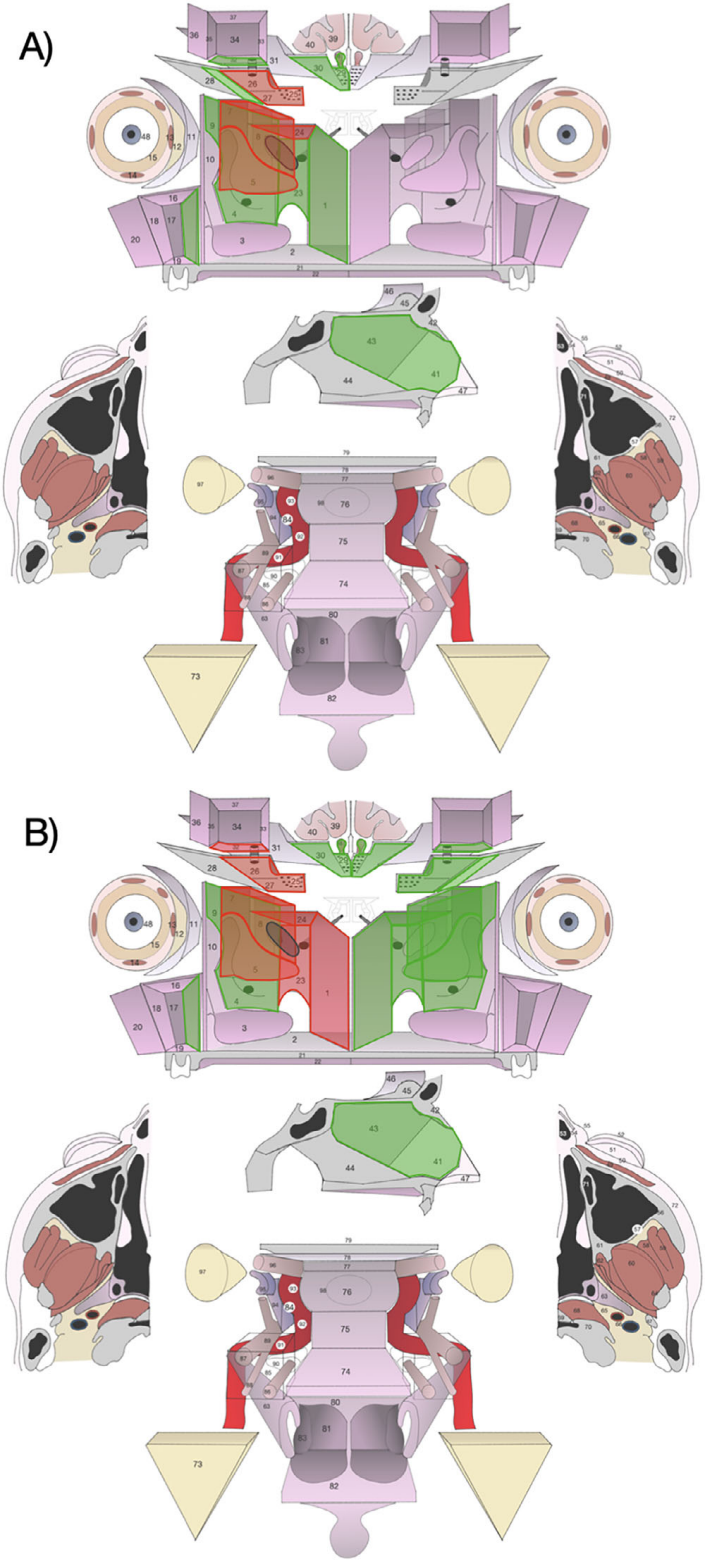
Examples of margin status assessment with the anatomic diagram after transnasal endoscopic surgery (TES). (A) “R0” resection, determined by the presence of non‐infiltrated structures (*green*) all around infiltrated structures (*red*), structures resected but not included in the pathological examination were marked with *grey* color. (B) “R1” resection, determined by the presence of infiltrated structures (*red*) not surrounded by non‐infiltrated structures (*green*), structures resected but not included in the pathological examination were marked with *grey* color.

Three different methods of interpretation of SLRD were applied: (1) *Pathological method*: SLRD was classified “R0” if the pathologist could not identify tumor cells involving the margins of the main surgical specimen, or “R1” if tumor cells were found at the margin of the main surgical specimen;[25] (2) *Multidisciplinary method*: following multidisciplinary discussion involving the surgeon who performed the surgical ablation, a head and neck radiologist, and a head and neck pathologist, SLRD was classified as “R0” if (a) tumor cells were not identified in the margins of the main surgical specimen, (b) or when additionally resected, non‐oriented tissues were uninvolved and could be localized adjacent to the involved margin(s), (c) or when additionally resected, anatomically oriented tissues originally adjacent to the involved margin(s) were cancer‐free in margins facing the resection bed; otherwise, SLRD was classified as R1; (3) *Interpretation of surgical margins through the anatomic diagram presented in this study*: SLRD was classified “R0” if infiltrated subunits were surrounded by non‐infiltrated resected tissues; if at least one infiltrated structure was present at the boundary of resection, then SLRD was classified as “R1” (Figure 2).

The indication for adjuvant RT was based on the multidisciplinary classification of SLRD, integrated with other information, including tumor stage, histological grade, N‐status, perineural invasion (PNI), lymphovascular invasion (LVI).

### 2.3 Statistical Analysis

Statistical analysis was performed with “R” and “RStudio” programs (https://www.R‐project.org/), version 4.3.1 (R Foundation for Statistical Computing, Vienna, Austria).

The primary outcomes of the prognostic analysis were time‐to‐local‐recurrence (TTLR), which is synonymous with “local control”, and time‐to‐recurrence (TTR), which is synonymous with “disease control”. Recurrence of disease was defined as event (considering only local recurrence for TTLR and recurrence of any type for TTR), whereas disease‐free alive status or death without evidence of disease were considered censor. The prognostic effect of the three SLRD assessment methods was tested using: (1) the Kaplan–Meier method, evaluating the association of SLRD (assessed with the three interpretative strategies) with TTLR and TTR, and (2) via the adjusted hazard ratio (HR) with 95% confidence interval (CI) calculated from a multivariable Cox proportional hazards model of TTLR and TTR built with a priori selection of the following covariates: age, pT category, histologic subtype, and adjuvant RT for ITAC patients; age, pT category, pN category, grading, and adjuvant RT for sinonasal SCC patients. In cases where model convergence was not achieved, the non‐converging covariate was incorporated as a stratification factor, allowing baseline hazards to vary across strata without estimating separate coefficients. Model assumptions and multicollinearity were checked, as appropriate. The discriminative capacity of local recurrence events was estimated through the difference of the three methods in terms of Harrell's concordance index (C‐index), the area under the receiver operating characteristic (ROC) curve (AUC), and the incremental AUC (iAUC), calculated from the aforesaid multivariable models.

## 3 Results

This study included 105 patients (78.1% males and 21.9% females), whose characteristics are described in Table 2. Among them, 50 (47.6%) were affected by SCC, and 55 (52.4%) by ITAC.

**TABLE 2:** General characteristics of the population.

		ITAC *N* (% OR IQ RANGE)		SCC *N* (% OR IQ RANGE)		Global *N* (% OR IQ RANGE)	
Number of patients		55		50		105	
Gender	Male	46 (83.6%)		36 (72.0%)		82 (78.1%)	
	Female	9 (16.4%)		14 (28.0%)		23 (21.9%)	
Median age at surgery (years)		66 (57.0–72.7)		60 (56.7–76.0)		66 (57.0–74.2)	
Epicenter	Nasoethmoid	55 (100.0%)		19 (38.0%)		74 (70.4%)	
	Maxilla	0 (0.0%)		23 (46.0%)		23 (21.9%)	
	Nasal fossa	0 (0.0%)		5 (10.0%)		5 (4.8%)	
	Nasal vestibule	0 (0.0%)		3 (6.0%)		3 (2.9%)	
PT Classification	T1	15 (27.3%)		11 (22.0%)		26 (24.8%)	
	T2	19 (34.5 %)		4 (8.0%)		23 (21.9%)	
	T3	4 (7.3%)		12 (24.0%)		16 (15.2%)	
	T4a	9 (16.4%)		15 (30.0%)		24 (22.9%)	
	T4b	8 (14.5%)		8 (16.0%)		16 (15.2%)	
N Classification	N0	55 (100.0%)		44 (88.0%)		99 (94.3%)	
	N1	0 (0.0%)		3 (6.0%)		3 (2.8%)	
	N2	0 (0.0%)		2 (4.0%)		2 (1.9%)	
	N3	0 (0.0%)		1 (2.0%)		1 (0.9%)	
Subtype / Grade		**Colic**	7 (12.7%)	**G1**	3 (6.0%)	−	−
		**Papillary**	11 (20.0%)	**G2**	12 (24.0%)	−	−
		**Solid**	5 (9.1%)	**G3**	29 (58.0%)	−	−
		**Mucinous**	20 (36.4%)	**Unknown**	6 (12.0%)	−	−
		**Unknown**	12 (21.8%)	−	−	−	−
Adjuvant treatment	No	18 (32.7%)		19 (38.0%)		37 (35.2%)	
	Yes	36 (65.5%)		31 (62.0%)		67 (63.7%)	
	Unknown	1 (1.8%)		0 (0.0%)		1 (0.9%)	
Median follow‐up (months)		33.0 (14.0–64.0)		27.0 (11.1–49.3)		30.0 (11.8–58.7)	

Abbreviations: ITAC, intestinal‐type adenocarcinoma; SCC, squamous cell carcinoma.

Table 3 shows the classification of ITAC and SCC patients in the “R0” and “R1” categories.

**TABLE 3:** Margin status classification according to the three interpretative methods.

	Method #1 (Pathological Interpretation)		Method #2 (Multidisciplinary Interpretation)		Method #3 (Anatomic Diagram‐Based Interpretation)	
	SCC	ITAC	SCC	ITAC	SCC	ITAC
**R0 (%)**	29 (27.6%)	41 (39.0%)	40 (38.1%)	46 (43.8%)	23 (21.9%)	38 (36.2%)
	70 (66.7%)		86 (81.9)		61 (58.1%)	
**R1 (%)**	21 (20.0%)	14 (13.3%)	10 (9.5%)	9 (8.6%)	27 (25.7%)	17 (16.2%)
	35 (33.3%)		19 (18.1%)		44 (41.9%)	

Abbreviations: ITAC, intestinal‐type adenocarcinoma; SCC, squamous cell carcinoma.

**FIGURE 3:**
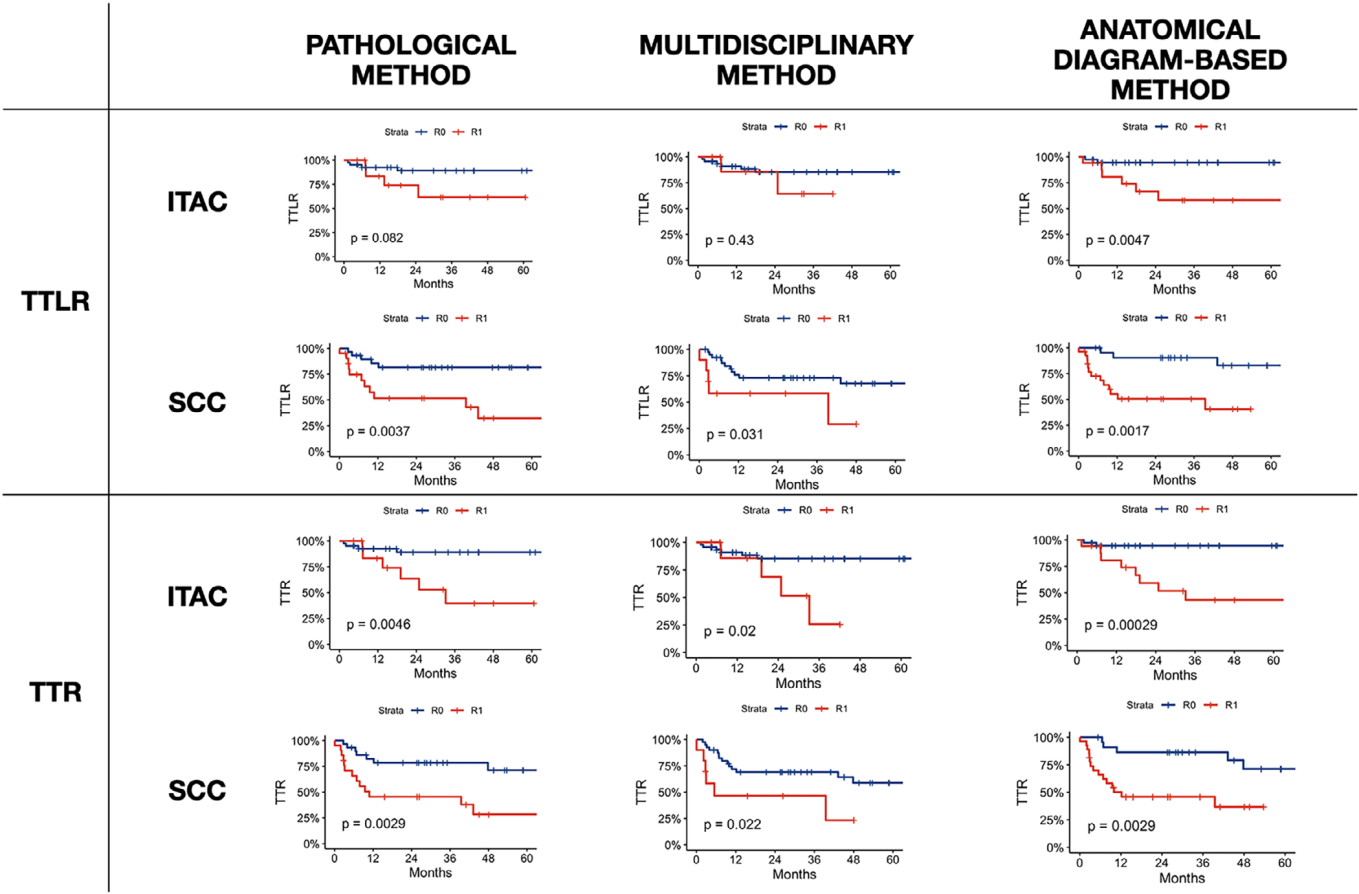
Kaplan–Meier curves estimating time‐to‐local‐recurrence (TTLR) and time‐to‐recurrence (TTR) for the 3 methods of interpretation of surgical margins in sinonasal squamous cell carcinoma (SCC) and nasoethmoidal intestinal‐type adenocarcinoma (ITAC) groups.

For both the ITAC and SCC cohorts, all three interpretative methods of the presence of SLRD demonstrated prognostic validity (Figure 3). The details of the 5‐year TTLR and TTR stratified for margin status according to the three SLRD inference methods are described in Table 4.

**TABLE 4:** 5‐year TTLR and TTR stratified for margin status according to the three SLRD inference methods.

		Method #1 (Pathological Interpretation)			Method #2 (Multidisciplinary Interpretation)			Method #3 (Anatomic Diagram‐Based Interpretation)		
		R0	R1	*p*	R0	R1	p	R0	R1	*p*
**TTLR**	**ITAC**	89.2%	61.7%	0.082	85.3%	64.3%	0.431	94.5%	58.2%	**0.005**
	**SCC**	81.6%	32.3%	**0.004**	67.8%	29.2%	**0.031**	82.9%	40.5%	**0.002**
**TTR**	**ITAC**	89.2%	39.7%	**0.005**	85.3%	25.7%	**0.019**	94.5%	43.1%	**< 0.001**
	**SCC**	71.3%	28.5%	**0.003**	58.9%	23.3%	**0.022**	71.5%	36.8%	**0.003**

Abbreviations: ITAC, intestinal‐type adenocarcinoma; SCC, squamous cell carcinoma.

When including SLRD in multivariable models, the anatomic diagram‐based SLRD was the only one independently associated with TTR for both ITAC and SCC cohorts after adjusting for potential confounders (Tables 5 and 6). In the ITAC group, histological subtype and adjuvant RT were non‐converging covariates in the multivariate model of TTLR and were considered in the model as a stratification factor.

**TABLE 5:** Multivariate model for 5‐year TTLR and TTR for the intestinal‐type adenocarcinoma (ITAC) population.

		Method #1 (Pathological Interpretation)			Method #2 (Multidisciplinary Interpretation)		Method #3 (Anatomical Diagram‐Based Interpretation)	
		HR (95% CI)		p	HR (95% CI)	p	HR (95% CI)	*p*
**TTLR**	**Margin status**	**R0**	Ref	0.643	Ref	0.652	Ref	0.272
		**R1**	0.68 (0.13–3.45)		0.66 (0.11–3.93)		2.63 (0.47–14.71)	
	**Age**		1.00 (0.94–1.06)	0.988	1.00 (0.95–1.07)	0.823	0.99 (0.93–1.06)	0.828
	**pT**	**1**–**2**	Ref	0.589	Ref	0.613	Ref	0.802
		**3‐4**	1.88 (0.19–18.37)		1.77 (0.19–16.12)		1.31 (0.15–11.20)	
**TTR**	**Margin status**	**R0**	Ref	0.907	Ref	0.667	Ref	**0.034**
		**R1**	1.08 (0.30–3.85)		1.32 (0.36–4.76)		5.46 (1.13–26.28)	
	**Age**		0.99 (0.94–1.04)	0.856	0.99 (0.93–1.04)	0.742	0.98 (0.91–1.03)	0.414
	Subtype*	**Low‐grade**	Ref	0.500	Ref	0.458	Ref	0.368
		**High‐grade**	2.04 (0‐25–16.29)		2.19 (0.27–17.50)		2.60 (0.32–20.94)	
	**pT**	**1**–**2**	Ref	**0.008**	Ref	**0.012**	Ref	0.094
		**3**–**4**	8.41 (1.74–40.55)		7.41 (1.53–35.73)		3.80 (0.79–18.26)	
	**Adjuvant treatment**	**No**	Ref	**< 0.001**	Ref	**< 0.001**	Ref	**0.015**
		**Yes**	0.04 (0.01–0.17)		0.03 (0.01–0.17)		1.37 (0.03–0.67)	

Abbreviations: CI, confidence interval; HR, hazard ratio.

*Low‐grade ITACs: colic and papillary subtypes, according to Barnes classification; high‐grade ITACs: solid and mucinous subtypes, according to Barnes classification.

**TABLE 6:** Multivariate model for TTLR and TTR adjusted for age, grading, pT, N‐status, and adjuvant treatment as covariates for the squamous cell carcinoma (SCC) population.

			Method #1 (Pathological Interpretation)		Method #2 (Multidisciplinary Interpretation)		Method #3 (Anatomical Diagram‐Based Interpretation)	
			HR (95% CI)	P	HR (95% CI)	p	HR (95% CI)	*p*
**TTLR**	**Margin status**	**R0**	Ref	0.090	Ref	0.101	Ref	**0.010**
		**R1**	2.87 (0.85–9.76)		2.71 (0.82–8.88)		6.55 (1.57–27.34)	
	**Age**		1.03 (0.98–1.08)	0.199	1.03 (0.98–1.08)	0.215	1.03 (0.99–1.08)	0.146
	**Grading**	**1**–**2**	Ref	0.787	Ref	0.843	Ref	0.513
		**3**	0.82 (0.21–3.23)		0.87 (0.23–3.36)		0.63 (0.16–2.51)	
	**pT**	**1**–**2**	Ref	0.065	Ref	0.070	Ref	0.317
		**3**–**4**	9.40 (0.86–102.15)		8.40 (0.84–84.08)		3.32 (0.32–34.98)	
	**Adjuvant treatment**	**No**	Ref	0.203	Ref	0.186	Ref	0.362
		**Yes**	0.41 (0.11–1.61)		0.39 (0.10–1.57)		0.53 (0.14–2.06)	
	**N Status**	**N0**	Ref	**0.032**	Ref	**0.010**	Ref	**0.002**
		**N+**	4.10 (1.13–14.93)		5.65 (1.50–21.23)		10.76 (2.34–49.50)	
**TTR**	**Margin status**	**R0**	Ref	0.092	Ref	0.092	Ref	**0.022**
		**R1**	2.42 (0.86–6.80)		2.50 (0.86–7.26)		3.78 (1.20–11.87)	
	**Age**		1.03 (0.99–1.07)	0.189	1.03 (0.99–1.07)	0.156	1.03 (0.99–1.07)	0.150
	**Grading**	**1‐2**	Ref	0.835	Ref	0.770	Ref	0.974
		**3**	1.15 (0.31–4.17)		1.21 (0.33–4.37)		0.98 (0.27–3.60)	
	**pT**	**1**–**2**	Ref	0.095	Ref	0.092	Ref	0.368
		**3**–**4**	4.68 (0.76–28.84)		4.55 (0.78–26.57)		2.27 (0.38–13.48)	
	**Adjuvant treatment**	**No**	Ref	0.334	Ref	0.275	Ref	0.507
		**Yes**	0.54 (0.15–1.89)		0.49 (0.14–1.76)		0.66 (0.19–2.22)	
	**N Status**	**N0**	Ref	0.058	Ref	**0.019**	Ref	**0.006**
		**N+**	3.25 (0.96–10.98)		4.36 (1.26–15.09)		6.55 (1.69–25.34)	

Abbreviations: CI, confidence interval; HR, hazard ratio.

**FIGURE 4:**
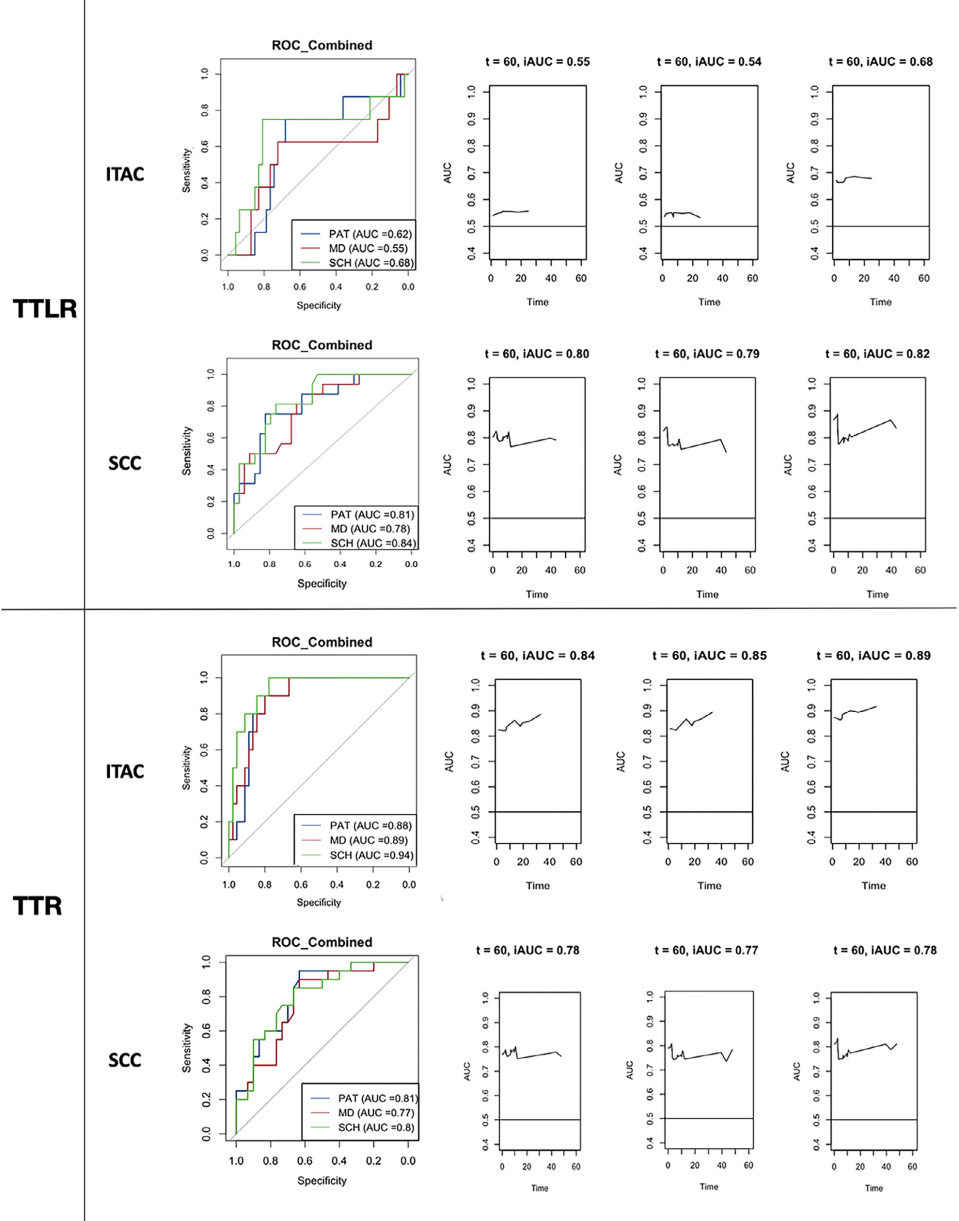
Discriminative capacity of the three subclinical local residual disease inference methods in sinonasal squamous cell carcinoma (SCC) and nasoethmoidal intestinal‐type adenocarcinoma (ITAC) groups.

Discriminative performance in predicting local recurrence of different models is reported in Table 7 and Figure 4. The C‐index for the model including the anatomic scheme‐based SLRD was higher compared to other methods, in both ITAC and SCC populations. AUC and iAUC resulted higher when inferring SLRD with the anatomic scheme‐based method in the ITAC cohort, while it was relatively independent of the method of SLRD inference in the SCC population (Table 7).

**TABLE 7:** C‐index, AUC, and 60‐month iAUC for the multivariate models for TTLR with the three inference methods of the interpretation of margins status.

		**C‐index**			**AUC**			**60‐month iAUC**		
		**Method #1**	**Method #2**	**Method #3**	**Method #1**	**Method #2**	**Method #3**	**Method #1**	**Method #2**	**Method #3**
**TTLR**	**ITAC**	0.58	0.54	0.62	0.62	0.55	0.78	0.55	0.54	0.68
	**SCC**	0.77	0.76	0.81	0.81	0.78	0.84	0.80	0.79	0.82
**TTR**	**ITAC**	0.81	0.80	0.86	0.88	0.89	0.94	0.84	0.85	0.89
	**SCC**	0.75	0.74	0.78	0.81	0.77	0.80	0.78	0.77	0.78

Abbreviations: AUC, area under the curve; C‐index, concordance index; iAUC, incremental area under the curve; ITAC, intestinal‐type adenocarcinoma; SCC, squamous cell carcinoma.

**FIGURE 5:**
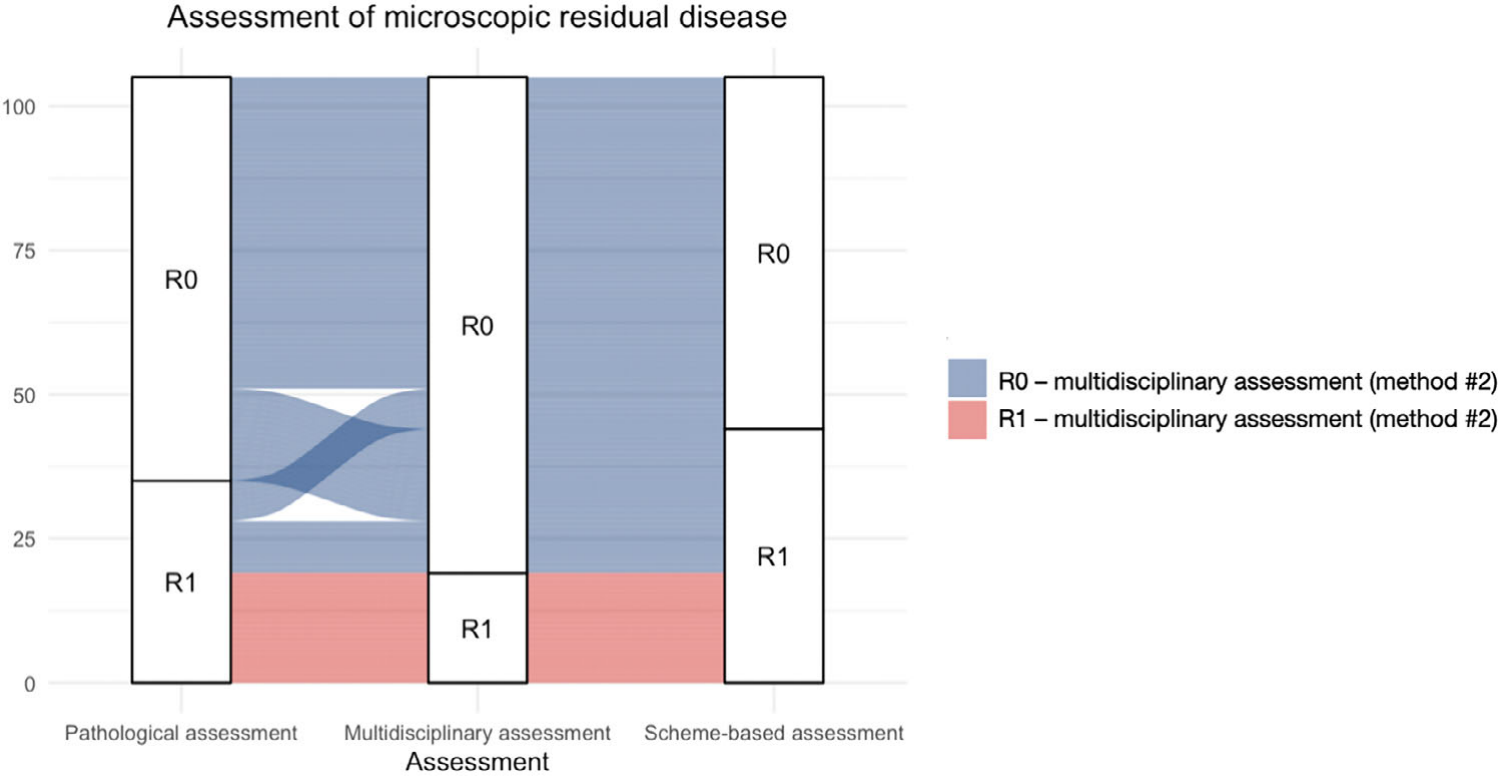
Alluvial plot showing the transition of surgical margin status of patients from the pathological method, the multidisciplinary method, and the anatomical diagram‐based method.

Using the anatomical diagram‐based method, the margin status was reclassified from R0 assessed through Method #2 (multidisciplinary interpretation) to R1 in 25 patients (23.8%). Among this subgroup of patients, 7 patients (28.0%) were not recommended adjuvant RT, comprising 6 of 50 patients with SCC (12.0%) and 1 of 55 patients with ITAC (1.8%). Of the SCC subgroup, 3/6 (50.0%) patients subsequently developed local recurrence and died of disease. No patient was reclassified from R1 category to R0 (Figure 5).

## 4 Discussion

TES is currently considered the gold standard surgical technique for most nasoethmoidal malignancies, when negative margins can be achieved. Achieving negative margins is the main surgical goal, as it ensures the highest chance of cure and represents the primary prognostic factor under surgeons’ control [26, 27]. However, the interpretation of surgical margins can be complex, and a multidisciplinary evaluation is essential to minimize potential biases, as demonstrated in previous experiences in the head and neck oncology field [28]. The multiblock resection strategy often required in TES further complicates the interpretation of margin status, making standardized pathological assessment even more critical [16]. Therefore, a comprehensive topographic analysis of the resected specimen from a surgical and pathological standpoint is crucial to understand the three‐dimensional extension of the tumor and to assess the presence of SLRD (i.e., R1) [18]. However, there is a lack of validated and universal systems for reporting intraoperative tissue sampling during TES, making SLRD estimation a non‐standardized and poorly reproducible medical process.

To improve the definition of tumor extension, margin status interpretation, and multidisciplinary communication, Bastier and de Gabory [24] ideated a three‐dimensional diagram of the sinonasal tract and neighboring anatomical structures, permitting easy visualization of resected and involved structures. In the present study, a modified version of such anatomic diagram (Figure 1) is presented and validated for SLRD inference in a cohort of 105 patients affected by sinonasal SCC and nasoethmoidal ITAC, comparing it with an exclusively pathological and a multidisciplinary approach. In our analysis, all three SLRD interpretative methods proved to be valid prognosticators in both ITAC and SCC patients. Nevertheless, the accuracy in predicting local recurrence was suboptimal, particularly for ITAC (Table 7). Anatomic diagram‐based SLRD was the only method demonstrating independent statistical association with risk of recurrence after adjusting for relevant confounders. Furthermore, the anatomic diagram demonstrated the highest discriminative capacity for recurrence events, particularly for ITAC. These results support the integration of the anatomical diagram in the interpretative process of SLRD. Of note, both TTLR and TTR were used as metrics to assess the accuracy of SLRD estimation, since subclinical localization of ITAC/SCC at the primary site can potentially explain any type of recurrence (local, regional, distant), although causality is certainly less controversial for TTLR. Using the anatomical diagram‐based method, margin status was reclassified from R0 to R1 in 25 patients (23.8%). This reclassification could have potentially influenced the indication for adjuvant RT, although the final decision regarding postoperative treatment is made within a multidisciplinary setting and considers multiple clinical and pathological factors. Among the 25 patients with upgraded margin status, 7 (28.0%) did not receive adjuvant treatment. These 7 patients might have received a different recommendation had the anatomical diagram‐based interpretation been adopted at the time. Of note, 3 of them developed local recurrence and experienced disease‐specific mortality.

Notably, the multidisciplinary evaluation method proved to be comparable or even slightly worse in predicting recurrence events than the exclusively pathological examination. A possible interpretation of this result could be related to confirmation and self‐enhancement bias; indeed, when the same surgeon who performed the procedure is involved in the margin status assessment, a higher rate of systematic type II error (i.e., false negatives) was observed. Surgeons might have tended to optimistically interpret orientation of tissue samples, increasing the rate of false negative SLRD. This aspect further reinforces the utility of relying on a standardized and validated method for SLRD assessment. Although the small sample size limits definitive conclusions, the anatomical diagram method would likely increase adjuvant RT indications. The anatomical diagram method might lead to some overtreatment, yet in the subgroup of patients who did not undergo adjuvant RT and had upgraded margin status, 3 of 7 (42.9%) might have avoided recurrence—or, at least, had SLRD treated—if RT had been recommended based on the diagram. Treatment decisions, however, remain multifactorial, considering tumor extension, nodal status, grade, and subtype (Table S1).

Several limitations should be considered in this study. First, the retrospective analysis is inherently subject to potential bias. Although patient data were collected according to institutional standards, some variability in surgical or pathological reports may have influenced the interpretation. Second, the relatively limited sample size may have reduced the statistical power of subgroup analyses as well as the generalizability of our findings.

Furthermore, the effectiveness of the anatomic diagram relies on accurate and detailed intraoperative mapping, which may vary depending on the complexity of each case. Lastly, while our results are promising, validation in larger, independent cohorts is necessary to confirm the reproducibility and clinical value of this method.

## 5 Conclusions

The present study suggested that inference of SLRD following TES for the most common sinonasal cancers is limited by suboptimal accuracy. The anatomic diagram presented in this study represents a potential strategy for improving SLRD assessment after TES performed for sinonasal SCC and ITAC. Indeed, this method offers superior stratification in terms of TTR compared to traditional approaches. This advancement might improve multidisciplinary communication and may contribute to a more precise overall management of patients affected by sinonasal cancers.

## Author Contributions

*Conceptualization*: Marco Ferrari, Stefano Taboni, Leonardo Calvanese, Piergiorgio Gaudioso, Giacomo Contro, Tommaso Saccardo, Gabriele Testa, Vittorio Rampinelli, Alberto Schreiber, Davide Mattavelli, Cesare Piazza, and Piero Nicolai. *Methodology*: Marco Ferrari, Davide Mattavelli, Leonardo Calvanese, Stefano Taboni, and Piergiorgio Gaudioso. *Formal analysis and investigation*: Piergiorgio Gaudioso, Leonardo Calvanese, Stefano Taboni, Giacomo Contro, Tommaso Saccardo, Gloria Schiavo, Diego Cazzador, Gabriele Testa, Vittorio Rampinelli, Alberto Schreiber, Davide Mattavelli, Cesare Piazza, Piero Nicolai, and Marco Ferrari. *Writing—original draft preparation*: Piergiorgio Gaudioso, Leonardo Calvanese, and Marco Ferrari. *Writing—review and editing*: Piergiorgio Gaudioso, Leonardo Calvanese, Stefano Taboni, Giacomo Contro, Tommaso Saccardo, Gloria Schiavo, Diego Cazzador, Vittorio Rampinelli, Alberto Schreiber, Davide Mattavelli, Cesare Piazza, Emanuelli Enzo, Piero Nicolai, and Marco Ferrari. *Supervision*: Marco Ferrari, Davide Mattavelli, Cesare Piazza, and Piero Nicolai.

## Supporting information




**Supporting File 1**: alr70071‐sup‐0001‐tablesS1‐S3.docx
