# Supplementary material for: Anatomic Diagram as a Novel Assessment Strategy for Subclinical Local Residual Disease in Sinonasal Squamous Cell Carcinoma and Intestinal‐Type Adenocarcinoma
Source: Int Forum Allergy Rhinol. 2025 Nov 28;16(4):333–44. doi: 10.1002/alr.70071 (PMC13047938; doi:10.1002/alr.70071)
Supplement: Supplementary file 1 — Supporting File 1: alr70071‐sup‐0001‐tablesS1‐S3.docx [file ALR-16-333-s001.docx]

**Supplementary Table 1**. Multivariable logistic regression model was constructed to evaluate the specific impact of variables on the indication to adjuvant radiotherapy.

| Variable | | SCC | | ITAC | | |
| --- | --- | --- | --- | --- | --- | --- |
|  |  | HR (95%CI) | p-value | HR (95%CI) | p-value | |
| Age | | 1.0 (0.1-9.2) | 0.843 | 0.9 (0.8-1.1) | | 0.090 |
| pT | Early stage | Ref | **<0.001** | Ref | 0.089 | |
|  | Advanced stage | 25.6 (4.6-231.3) |  | 7.7 (0.9-167.8) |  |  |
| cN | N0 | Ref | 0.335 | - | | - |
|  | N+ | 0.3 (0.1 - 3.4) |  | - | |  |
| Grade | G1-2 | Ref | 0.647 | - | | - |
|  | G3 | 0.6 (0.1-3.8) |  | - | |  |
| Subtype | Low-grade | - | - | Ref | | 0.259 |
|  | High-grade | - |  | 2.8 (0.5-19.6) | |  |
| Margins (Multidisciplinary method - #2) | R0 | Ref | 0.175 | Ref | | 0.994 |
|  | R1 | 5.2 (0.6-9.2) |  | 16.4 (2.0-78.4) | |  |

**Supplementary Table 2**. 5-year TTLR and TTR stratified for margin status according to the three MRD inference methods in patients with follow-up longer than 1 year.

|  | | **METHOD #1**  **(PATHOLOGICAL INTERPRETATION)** | | | **METHOD #2**  **(MULTIDISCIPLINARY INTERPRETATION)** | | | **METHOD #3**  **(ANATOMIC DIAGRAM-BASED INTERPRETATION)** | | |
| --- | --- | --- | --- | --- | --- | --- | --- | --- | --- | --- |
|  |  | **R0** | **R1** | **p** | **R0** | **R1** | **p** | **R0** | **R1** | **p** |
| **TTLR** | **ITAC** | 93.4 | 66.7 | **0.041** | 91.1 | 64.3 | 0.124 | 96.5 | 66.6 | **0.013** |
|  | **SCC** | 91.3 | 47.6 | **0.022** | 83.3 | 35.7 | **0.020** | 91.6 | 55.5 | **0.008** |
| **TTR** | **ITAC** | 93.4 | 42.9 | **<0.001** | 91.1 | 25.7 | **0.001** | 96.5 | 49.4 | **<0.001** |
|  | **SCC** | 83.0 | 43.3 | **0.014** | 76.3 | 28.6 | **0.004** | 82.5 | 51.8 | **0.010** |

**Supplementary Table 3**. 5-year TTLR and TTR stratified for margin status according to the three MRD inference methods in patients with follow-up longer than 2 years.

|  | | **METHOD #1**  **(PATHOLOGICAL INTERPRETATION)** | | | **METHOD #2**  **(MULTIDISCIPLINARY INTERPRETATION)** | | | **METHOD #3**  **(ANATOMIC DIAGRAM-BASED INTERPRETATION)** | | |
| --- | --- | --- | --- | --- | --- | --- | --- | --- | --- | --- |
|  |  | **R0** | **R1** | **p** | **R0** | **R1** | **p** | **R0** | **R1** | **p** |
| **TTLR** | **ITAC** | 96.0 | 71.4 | **0.008** | 96.3 | 60.0 | **0.008** | 100.0 | 70.0 | **0.007** |
|  | **SCC** | 95.0 | 56.2 | 0.056 | 89.1 | 40.0 | **0.015** | 91.7 | 64.6 | **0.044** |
| **TTR** | **ITAC** | 96.0 | 53.6 | **0.004** | 96.3 | 30.0 | **<0.001** | 100.0 | 58.3 | **0.001** |
|  | **SCC** | 86.3 | 50.0 | **0.036** | 81.7 | 30.0 | **0.003** | 82.5 | 58.2 | **0.042** |
